# Breakthrough for Trauma Treatment: Safety and Efficacy of MDMA-Assisted Psychotherapy Compared to Paroxetine and Sertraline

**DOI:** 10.3389/fpsyt.2019.00650

**Published:** 2019-09-12

**Authors:** Allison A. Feduccia, Lisa Jerome, Berra Yazar-Klosinski, Amy Emerson, Michael C. Mithoefer, Rick Doblin

**Affiliations:** ^1^Department of Research Development and Regulatory Affairs, MAPS Public Benefit Corporation, Santa Cruz, CA, United States; ^2^Multidisciplinary Association for Psychedelic Studies, Santa Cruz, CA, United States; ^3^MAPS Public Benefit Corporation, Santa Cruz, CA, United States; ^4^Department of Psychiatry and Behavioral Sciences, Medical University of South Carolina, Charleston, SC, United States

**Keywords:** methylenedioxymethamphetamine, posttraumatic stress disorder, breakthrough therapy, sertraline, paroxetine, anxiety

## Abstract

Unsuccessfully treated posttraumatic stress disorder (PTSD) is a serious and life-threatening disorder. Two medications, paroxetine hydrochloride and sertraline hydrochloride, are approved treatments for PTSD by the Food and Drug Administration (FDA). Analyses of pharmacotherapies for PTSD found only small to moderate effects when compared with placebo. The Multidisciplinary Association for Psychedelic Studies (MAPS) obtained Breakthrough Therapy Designation (BTD) from the FDA for 3,4-methylenedioxymethamphetamine (MDMA)-assisted psychotherapy for treatment of PTSD on the basis of pooled analyses showing a large effect size for this treatment. This review covers data supporting BTD. In this treatment, MDMA is administered with psychotherapy in up to three monthly 8-h sessions. Participants are prepared for these sessions beforehand, and process material arising from the sessions in follow-up integrative psychotherapy sessions. Comparing data used for the approval of paroxetine and sertraline and pooled data from Phase 2 studies, MAPS demonstrated that MDMA-assisted psychotherapy constitutes a substantial improvement over available pharmacotherapies in terms of safety and efficacy. Studies of MDMA-assisted psychotherapy had lower dropout rates compared to sertraline and paroxetine trials. As MDMA is only administered under direct observation during a limited number of sessions, there is little chance of diversion, accidental or intentional overdose, or withdrawal symptoms upon discontinuation. BTD status has expedited the development of MAPS phase 3 trials occurring worldwide, leading up to a planned submission seeking FDA approval in 2021.

**Clinical Trial Registration:** www.ClinicalTrials.gov, identifiers NCT00090064, NCT00353938, NCT01958593, NCT01211405, NCT01689740, NCT01793610.

## Introduction

Breakthrough therapy designation (BTD) is one of the Food and Drug Administration’s (FDA) expedited drug development pathways. To be eligible for BTD, a sponsor must demonstrate that the investigational product is intended to treat a serious and life-threatening condition, with preliminary evidence supporting a substantial advantage at a clinically significant endpoint over existing drugs (1). On August 16, 2017, the FDA granted breakthrough therapy designation for MDMA-assisted psychotherapy for the treatment of posttraumatic stress disorder (PTSD). This application was among the 45% of applications granted BTD status in 2017 (2). The aim of this review is to summarize the data and rationale presented in the application that led FDA to grant this designation.

PTSD is considered a serious and life-threatening disorder and is associated with increased mortality, cardio-metabolic morbidity, and suicide risk. PTSD negatively impacts a person’s daily life, often resulting in fractured relationships, depression, decreased daily functioning, diminished cognitive and psychosocial functioning, substance abuse, and high-cost healthcare utilization ($34.9 billion in inflation-adjusted charges for hospitalizations (2002–2011) (3). Approximately 7% of the U.S. population, and 11.2–17.1% of veterans (4), will have PTSD sometime in their life (5).

Only two drugs, the selective serotonin reuptake inhibitors (SSRIs) sertraline hydrochloride (Zoloft) and paroxetine hydrochloride (Paxil), are approved oral medications for PTSD (6–8). These medications and trauma-focused psychotherapies (e.g., eye movement desensitization, cognitive processing therapy, prolonged exposure) are recommended as first-line treatments for PTSD (9–12). In a meta-analysis evaluating psychotherapy versus pharmacotherapy, trauma-focused psychotherapies resulted in greater and longer lasting improvements than medications (12). Meta-analyses and network meta-analyses found paroxetine, but not sertraline, performed better than placebo (13, 14). Hoskins and colleagues reported that SSRIs had a small effect size with respect to PTSD symptom reduction. When compared to a control group, SSRIs either had insignificant effects or small/moderate effects, while trauma-focused therapies varied from small to large effects (12). The average dropout rate for the 55 studies included in the meta-analysis was 29% (0–79%) demonstrating that many individuals fail to tolerate or respond to available treatments (12), including trauma-focused psychotherapies, where the dropout can range from 28 to 68% (15, 16). A network meta-analysis reported that dropout rate for paroxetine and sertraline was greater than placebo (14).

The Multidisciplinary Association for Psychedelic Studies (MAPS) holds an Investigational New Drug Application (IND) for MDMA as an adjunct to psychotherapy for treatment of PTSD. MAPS has sponsored six phase 2 trials of MDMA-assisted psychotherapy for PTSD that lasted from April 2004 to March 2017. The safety and efficacy results from these trials were submitted to the FDA, along with a summary of the sertraline and paroxetine data that supported the New Drug Application (NDA) for approval of these drugs for the indication of PTSD. Sertraline and paroxetine summary data was extracted from documents found in the FDA drug database, including the Review and Evaluation of Clinical Data and the drug labels (17–20).

Here, we present the evidence included within the breakthrough therapy application showing that MDMA-assisted psychotherapy was superior in phase 2 trials in terms of safety and efficacy compared to the two approved SSRIs for treatment of PTSD. The control groups in the MDMA trials also received intensive psychotherapy (approximately 30 h), while SSRIs pivotal trials used a placebo without any type of therapy for comparison. Since the FDA does not regulate psychotherapy, the BT application did not compare MDMA-assisted psychotherapy to trauma-focused therapies. However, since trauma-focused therapies have evidence for the greatest effectiveness in reducing PTSD symptoms, we have included an additional section in this review comparing MDMA-assisted-psychotherapy with first-line psychological therapies.

## Efficacy and Durability of Response: MDMA vs. SSRIs

### MDMA-Assisted Psychotherapy

MDMA is a ring-substituted phenethylamine that is classified as an entactogen in the Merck Index (21) due to its properties that can promote empathy and compassion for self and others. MDMA stimulates release of serotonin, norepinephrine and dopamine, and may act directly on some adrenergic, cholinergic, and serotonergic receptors (22). MDMA elevates levels of the neurohormone oxytocin, an effect likely mediated through direct or indirect action on 5HT1A, 5HT2A, and 5HT4 receptors (23–25), as well as elevating levels of prolactin, arginine vasopressin (AVP), adrenocorticotrophic hormone (ACTH), and cortisol (26–29). MDMA possesses a unique pharmacodynamic profile in humans that includes increased emotional empathy, an increase in feelings of interpersonal closeness, greater prosocial behavior, and an increased ability to tolerate distressing memories, greater reward from pleasant memories, and less distress in response to social exclusion (30–34). Imaging studies found that MDMA reduced activity in brain areas associated with anxiety, including the amygdala, and increased activity in prefrontal cortex (35–37). Hypotheses for MDMA’s therapeutic action include enhanced fear extinction, memory reconsolidation, enhanced therapeutic alliance, widening a window of tolerance for distressing thoughts or experiences, and re-opening or enhancing a critical period for experiencing social reward (25, 38, 39). It is likely through these effects that MDMA augments and enhances effectiveness of psychotherapy.

Investigators have developed standardized psychotherapeutic methods for combining MDMA and psychotherapy that include up to 3 sessions with MDMA and up to 12 non-drug sessions. During preparatory sessions participants meet with the two co-therapists, usually one male and one female, when they discuss their goals, and concerns, and learn what to expect during the MDMA-assisted session. The psychotherapy during MDMA-assisted sessions is relatively non-directive, supporting the participants spontaneous experience, and designed to facilitate processing of challenging emotions in a safe and controlled setting (40–44). Participants may use eye shades, and may listen to a program of music designed to support the therapy. Periods of inner focus alternate with periods of talking to the therapists. Vital signs are assessed periodically. Material arising during MDMA-assisted psychotherapy sessions is integrated in subsequent psychotherapy visits. Subsequently, participants are encouraged to make time to explore and express their unfolding experience using journaling or artwork. Participants in Phase 2 studies were contacted for 7 days after each experimental session. More information concerning MDMA-assisted psychotherapy can be found in publications and in the MDMA Treatment Manual (42). Studies with a long term follow up demonstrate durable improvement in PTSD (41, 43–45), social anxiety in autistic adults (46), and anxiety associated with facing a life threatening illness (22, 38).

### Phase 2 Trials of MDMA-Assisted Psychotherapy for PTSD Treatment

The six Phase 2 studies of MDMA-assisted psychotherapy that supported the breakthrough application followed a randomized, double-blind, placebo-controlled design with the Clinician-Administered PTSD Scale for DSM-IV (CAPS-IV) as the primary efficacy measure (41, 44, 45, 47, 48). The CAPS-IV is an established measure of PTSD symptoms (49, 50). To enroll, participants were required to have a CAPS-IV total severity score of 50 or greater and to have failed to respond to or tolerate at least one course of treatment. The average duration of PTSD was 17.9 years. The basic study design for the six studies included three preparatory psychotherapy sessions, followed by 2–3 blinded, 8-h experimental psychotherapy sessions with MDMA (75–125 mg) or comparator/placebo (0–40 mg MDMA), and three 90-min non-drug integrative psychotherapy visits following each experimental session. Experimental sessions were scheduled approximately a month apart. Independent Raters (not present during treatment, blinded to group assignment) administered CAPS-IV at baseline, primary endpoint (3–8 weeks after two blinded sessions, or after three sessions in one study), and secondary endpoints (time points during the open-label crossover and at the 12-month follow-up).

Data was pooled across the six phase 2 studies (**Table 1**). Results showed that the active dose group (MDMA 75–125 mg, n = 72) was statistically superior to the control group (0–40 mg, n = 31) at the primary endpoint (independent samples t-test, p < 0.001), with average (SD) drop in CAPS-IV total scores −37.8 (29.29) for the active group and −11.6 (17.93) for the control group. There was large between-group Cohen’s *d* effect size (0.9).

**Table 1:** Pooled CAPS-IV data from six phase 2 MAPS-sponsored studies of MDMA-assisted psychotherapy.

	Active group (MDMA 75–125 mg) N = 72	Control group (MDMA 0–40 mg) N = 31
Change in CAPS-IV total scores^a^, mean (SD)	−37.8 (29.29)	−11.6 (17.93)
Cohen’s *d* effect size^b^	1.5	0.6
Dropouts, n (%)^c^	5 of 74 (6.8%)	3 of 31 (9.7%)

a Change in CAPS-IV scores from baseline to the primary endpoint (1–2 months post 2–3 MDMA sessions).

b Within-group Cohen’s d effect size calculated by dividing the change from baseline to primary endpoint by the standard deviation.

c For the active group, 3 terminated early but completed an endpoint assessment and 2 terminated early with no endpoint assessments. For the control group, 3 terminated early but completed an endpoint assessment.

Prior to enrollment in MAPS-sponsored Phase 2 trials, 17 and 35 subjects (of n = 105) had previously taken paroxetine and sertraline, respectively (**Table 2**). Twelve participants had tried both SSRIs. These individuals did not reach adequate symptom reduction or failed to tolerate the SSRIs. From this subset, 20/38 (52.6%) subjects that received active doses of MDMA (75–125 mg) no longer met criteria for PTSD at the primary endpoint. The average drop in CAPS-IV total scores was −40.1 (25.66) for participants who had previously taken paroxetine and −35.04 (27.5) in participants who had previously taken sertraline (**Table 2**). The other 14 subjects were randomized to the control group. The high response rate and large drops in CAPS-IV total score in this subset suggests that MDMA therapy may be able to effectively treat PTSD in individuals who do not adequately respond to SSRIs.

**Table 2:** Mean change from baseline to the primary endpoint in CAPS-IV total scores in MAPS-sponsored phase 2 subjects who had previously taken sertraline, paroxetine, or both.

	Paroxetine n = 17	Sertraline n = 35	Paroxetine/sertraline n = 12
Control group, mean (SD) (MDMA 0–40 mg)	−21.0 (24.01) n = 4	−15.9 (16.87) n = 10	−30.3 (18.50) n = 3
Active group (MDMA 75–125 mg)	−40.1 (25.66) n = 13	−35.04 (27.5) n = 25	−38.2 (29.90) n = 9

### Sertraline Phase 3 Trials for PTSD

Sertraline was investigated by Pfizer for treatment of PTSD in four studies of similar design with a 12-week flexible dose (50, 100, 150, and 200 mg with 25 mg starting dose for titration) (17, 20). Subjects who met DSM-III-R criteria with a CAPS-2 total score of 50 or greater were enrolled. Patients had a mean duration of PTSD for 12 years and 44% of patients also had a depressive disorder. Two of the four studies failed to find a significant difference between the sertraline and placebo treated groups on any of the primary efficacy outcomes. One study (640, n = 208) reported efficacy on CAPS-2 total score at week 12 [last observation carried forward (LOCF) method, p = 0.043] but not week 12 [observed case (OC)] or any earlier weeks. Placebo-subtracted effect size was 0.31, with a 6.8 point mean difference between groups in CAPS-2 total score (LOCF). The other study (671, n = 183) detected efficacy (OC) of sertraline at weeks 2 (p = 0.041), 4 (p = 0.0002), 6 (p = 0.011), 8 (p = 0.006), 10 (p = 0.04), and 12 (p = 0.016) on CAPS-2 but only in females which was influenced by mood improvement.

A combined analysis of the two positive studies found a significant difference between sertraline and placebo groups only in women but not in men. Results suggest much of the effect on PTSD scales correlated with improvement in the HAM-D, therefore it is unclear whether sertraline treats PTSD or comorbid depression, an indication the drug was already approved for. The report stated that there was insufficient evidence to support any efficacy claim beyond 3 weeks of treatment. However, a longer-term study that randomized responders (n = 96) in a 24-week open-label continuation trial of sertraline (50–200 mg/day), or switched to placebo for 28 weeks, found significantly reduced relapse rates for the sertraline group, in both males and females.

### Paroxetine Phase 3 Trials for PTSD

Paroxetine (20–50 mg/day) demonstrated superiority over placebo on change from baseline for the CAPS-2 total score in two multicenter, placebo-controlled studies in adults who met DSM-IV criteria for PTSD. The trials were sponsored by GlaxoSmithKline (18, 51). In these studies, 858 patients had PTSD symptoms with duration on average of 13 years. Major depressive disorder was present in 41% of patients and non-PTSD anxiety disorder was reported for 40% of patients. Primary outcomes were change from baseline to endpoint on CAPS-2 total score and the proportion of responders assessed by the Clinical Global Impression-Global Improvement Scale (CGI-I), a 3-item observer-rated scale.

In Study 1 (20 and 40 mg) and Study 2 (20 and 50 mg), paroxetine was significantly superior to placebo on both outcome measures. In Study 1 (n = 551), paroxetine was better than placebo (p < 0.001) at 4, 8, and 12-week time points for the LOCF and OC analyses. 71% of 40 mg paroxetine and 76% of 20 mg paroxetine treated patients met response criteria on CGI-I compared to 48% of placebo (p < 0.001). The difference between paroxetine and placebo groups on CAPS-2 total score was approximately 14 units for LOCF and OC analyses for both dose groups. In Study 2 (n = 307), paroxetine was better than placebo (p < 0.001) at 12-week time point for the LOCF and OC analyses. 76% of paroxetine treated patients met response criteria on CGI-I compared to 50% of placebo (p < 0.001). The difference between paroxetine and placebo groups on CAPS-2 total score was approximately 11 units for LOCF and 14 units for OC.

A third study with flexible doses (20–50 mg) found paroxetine to be significantly better than placebo on CAPS-2 total score, but not on CGI-I responders (defined as patients having a score of 1 “very much improved” or 2 “much improved”). In Study 3 (n = 322), CAPS-2 total score was statically superior in paroxetine group compared to placebo for LOCF (p = 0.047) but not OC analysis (p = 0.071) at the 12-week time point. On the CGI-I, 60% of paroxetine treated subjects met response criteria compared to 52% of placebo (not statistically significant). The difference between paroxetine and placebo groups on CAPS-2 total score was approximately 6 units for LOCF and OC analysis. Analyses did not detect any differences in gender on treatment outcomes.

The difference in CAPS-2 total scores between paroxetine and placebo in mean change from baseline at 12 weeks was roughly 6-14 units across the three studies. According to the drug label, the efficacy of paroxetine to treat PTSD beyond 12 weeks had not been investigated in controlled clinical trials, yet PTSD is a chronic condition.

### Comparison: SSRIs vs. MDMA

Primary efficacy evaluation of six MAPS-sponsored phase 2 trials on change from Baseline to Primary Endpoint in CAPS-IV Total Severity indicated a significant effect of MDMA over the comparator group (p < 0.001), with a large between-group effect size (0.9 Cohen’s *d* effect size) that was approximately double that of paroxetine (0.45–0.56) and triple that of sertraline (0.31–0.37). In comparison of mean change in CAPS total scores, placebo subtracted scores for sertraline ranged from 6.8–9.8 units, for paroxetine 6–14 units, and for MDMA 26.2 units (**Table 3**). The fact that the control group in MDMA studies received the same intensive psychotherapy as the active dose group adds to the clinical significance of these differences. Results from MAPS-sponsored MP-1 study detected significant (p = 0.013) difference between MDMA (125 mg) and placebo groups on CAPS-IV total scores 3–5 days after the first experimental session, demonstrating a rapid clinical response after a single MDMA dose. SSRIs require at least 2 weeks of daily dosing with dose titrations to produce any detectable PTSD symptom improvements, and one pivotal study of sertraline and one of paroxetine did not find significant improvement until after 12 weeks of daily drug administration. The beneficial effects of MDMA-assisted psychotherapy have been shown to last for at least 12 months in many participants (67.8% of n = 90 did not meet diagnostic criteria), while paroxetine (12 weeks) and sertraline (3 weeks) drug labels specify that long-term efficacy was not assessed. Sertraline was only shown to statistically significant in women and not men, while MDMA has been effective for both males and females with no difference in response measured

**Table 3:** Comparison of sertraline, paroxetine, and MDMA mean CAPS reduction LOCF, intent-to-treat.

	Sertraline		Paroxetine		MDMA	
	CAPS-2 (sertraline–placebo)^a^	Dropout %	CAPS-2 (paroxetine–placebo)^a^	Dropout %	CAPS-IV (MDMA– control)^b^	Dropout %
Study 1	−6.8 (effect size 0.31)	29.3%	−14 (effect size 0.56)	35.5%	−26.2 (effect size 0.9)	7.6%
Study 2	−9.8 (effect size 0.37)	28.4%	−11 (effect size 0.45)	39.0%	—	
Study 3	—		−6 (effect size 0.09)	33.0%	—	

a Effect sizes were not reported in FDA statistical package for paroxetine. Placebo subtracted effect. Size were determined from CAPS scores by calculating the change from baseline divided by the standard deviation.

b Primary endpoint was 1–2 months after 2–3 blinded experimental sessions.

Sertraline and paroxetine demonstrated superiority on the CAPS-2 over placebo in two 12-week pivotal trials which led to a new marketing label for the indication of PTSD. Both had small to medium placebo-subtracted effect sizes (0.31–0.37 and 0.45–0.56, respectively) and require daily dosing for 12 weeks.

## Compliance and Safety: MDMA vs. SSRIs

The dropout rate in active (75–125 mg blinded) MDMA-treated subjects in MAPS-sponsored Phase 2 trials was 6.8% (5 of 74, with 2 excluded for missing outcome data and 3 excluded for early termination, with outcome data), considerably less than SSRI trials where dropout rates were 11.7% in paroxetine-treated and 28% in sertraline-treated subjects, indicating that MDMA is better tolerated by a PTSD population than the two SSRIs. Reduced drop-out rates in MAPS’ Phase 2 studies may result from a strong therapeutic alliance, and commitment to the course of psychotherapy, as well as the therapeutic effects of MDMA. On the other hand, dropout rates (3 of 31, 9.7%) were also low for the control group which could reflect some benefit from the psychotherapy alone, or increased motivation to remain in the study to receive active MDMA during the open-label crossover segment.

In paroxetine trials, the most common adverse events (5% or greater and at least 2× that of placebo) in the PTSD population were: asthenia, sweating, nausea, dry mouth, diarrhea, decreased appetite, somnolence, libido decreased, abnormal ejaculation, female genital disorders, and impotence. Reported by 19% of subjects, nausea was the most frequently experienced treatment-emergent adverse event. For sertraline, the most common effects were nausea, headache, insomnia, diarrhea, dry mouth, ejaculation failure, somnolence, dizziness, and fatigue.

Administering MDMA in single doses spaced a month apart in a controlled setting has several inherent benefits over chronic daily dosing of paroxetine or sertraline. Firstly, compliance is not an issue in studies of MDMA, because all dosing occurs in a clinic under supervision, whereas SSRIs rely on patients self-administering daily doses which can be a challenge due to cognitive and behavioral impairments that can accompany PTSD (52).

Secondly, fewer side effects are reported after MDMA due to the limited number of administrations. Phase 2 safety data showed that reactions were reported most frequently on the day of MDMA administration and typically diminished in the few days following. The most commonly reported reactions on the day of the experimental session were anxiety, tight jaw/jaw clenching, lack of appetite, headache, and fatigue (48). On the day of blinded experimental sessions, reactions reported by the active MDMA group by at least 2x of the frequency of the control group were diarrhea, difficulty concentrating, dizziness, heavy legs, impaired gait/balance, jaw clenching/tight jaw, lack of appetite, nausea, nystagmus, paresthesia, perspiration, sensitivity to cold, thirst, and weakness. These findings are in line with clinical trials in healthy controls (53, 54). On the other hand, patients taking paroxetine and sertraline experience more prolonged adverse reactions due to steady state drug plasma levels across the 12-week treatment period.

Discontinuation of paroxetine and sertraline may be accompanied by adverse effects (55), likely caused by neuroadaptations of decreased levels of serotonin transporters in neuronal membranes after use of SSRIs (56). For discontinuation of sertraline and paroxetine gradual tapering is recommended, and patients should be monitored for discontinuation emergent symptoms, which can be very troubling. Adverse events during discontinuation (incidence of 2% or greater for paroxetine and at least 2x that of placebo) were abnormal dreams, paresthesia, and dizziness, and for sertraline, they were nausea, insomnia, and diarrhea (18, 20). Post-marketing surveillance identified a number of additional discontinuation emergent negative effects, including sensory disturbances, agitation, anxiety, nausea, and sweating; however causal relationship to drug hasn’t been confirmed.

Single doses of MDMA have not produced discontinuation symptoms. Some adverse reactions are reported during the 7 days following an MDMA dose, including anxiety, dizziness, depressed mood, fatigue, headache, jaw clenching or tightness, lack of appetite, nausea, and panic attack (48). By Day 5, the only reactions reported in over 20% of active dose participants were fatigue and anxiety. Both were reported by nearly equal numbers of active and control dose participants. Symptoms were mild to moderate in severity, and nearly all resolved within 7 days of dosing. Eight participants in the active dose group and three in the control group, reported a reaction on the seventh day of follow-up (not seven consecutive days of experiencing the reaction) that was therefore recorded as an adverse event (AE). Reactions fitting AE criteria and reported by more than two participants were anxiety and low mood, occurring in both active and control groups. Both are prominent symptoms of PTSD. Only three participants had the same reaction on day of experimental session and 7 days following the session, which included anxiety, low mood, and muscle tension.

Estimating risk of long-term deleterious effects of discrete doses of MDMA in a controlled setting compared to retrospective studies in people reporting ecstasy use is inappropriate for several reasons. Ecstasy can contain an unknown quantity of MDMA and adulterants, or no MDMA at all, and most people ingesting MDMA are polydrug users. Most studies are retrospective, with only a single prospective study reported detecting signs of a specific impairment in verbal memory in a sample of people reporting nonmedical use, without detecting any functional or structural changes in the brain (57, 58). Systematic reviews of the literature found that most research enrolls people whose lifetime use far exceeds the average (59–61). In contrast, cognitive function in three trials of MDMA-assisted psychotherapy failed to find impairment after any dose of MDMA (48). When asked about ‘ecstasy’ use at 12-month follow-up after participation in a Phase 2 trial, eight participants, six of whom had taken ecstasy prior to enrollment, reported having used it one to three times. This indicates that MDMA given in the context of psychotherapy does not have high abuse liability (41, 43, 44, 47, 62).

An additional risk of SSRIs is that they are contraindicated with MAOIs and some other drugs due to inhibition of P450 enzymes. Since these drugs are take-home medications, patients are at risk of accidentally consuming a contraindicated medication that could have serious adverse effects, including death. Accidental and intentional overdoses have been reported with both SSRIs (63). Since clinicians collect concomitant medication information at each session before administering MDMA, the risk for accidental use of a contraindicated medication is far reduced, and risk of overdose is eliminated by dispensing only the recommended dosage by a prescribing physician. Both SSRI drug labels state that alcohol is not recommended, but given that a significant number of people with PTSD also have comorbid alcohol use disorders, refraining from alcohol may be particularly problematic for this population and lead to negative effects (64, 65).

MDMA-assisted psychotherapy received BTD based on its use in treating PTSD, a serious and life-threatening condition, and on the basis of phase 2 clinical data that MDMA produced substantial clinical improvement and greater compliance than the two approved drugs for PTSD, paroxetine and sertraline. Data from Phase 2 provides evidence that PTSD, independent of cause, is treatable with 2 to 3 sessions of MDMA-assisted psychotherapy, and offers a larger treatment effect, increased compliance and lower risk of dropout, reduced possibility of drug interactions compared to paroxetine and sertraline. There have been no deaths related to MDMA in controlled Phase 1 and 2 studies, and if it is approved for clinical use, MDMA will be administered directly to patients, and only in licensed MDMA clinics under controlled conditions similar to those in clinical research. The single-dose regimen of MDMA produces fewer, self-limiting, transient side effects and greater compliance compared to daily dosing of paroxetine and sertraline.

## Comparison of MDMA-Assisted Psychotherapy vs. Trauma-Focused Therapies

In meta-analyses comparing efficacy of PTSD treatments investigated in randomized controlled trials, trauma-focused psychotherapies generally result in greater and more sustained response than pharmacotherapies and other psychological therapies (12, 66). Lee et al. report comparative effect sizes from meta-analyses of randomized trials that included a control condition, with controls for psychotherapy trials including supportive psychotherapy, biofeedback, and relaxation training, and excluding those with waitlist and treatment-as-usual controls. Compared to control, after 14–27 weeks of trauma-focused therapies the effect size was −0.96. For all medications, which included SSRIs, SNRIs, antiepileptics, antipsychotics, the effect size was −0.44. The magnitude of effect (0.9) of MDMA-assisted psychotherapy is in the range of first-line trauma-focused therapies. MDMA was compared to psychotherapy alone, or low dose MDMA plus psychotherapy, as the control condition and Phase 2 studies enrolled only participants who had previously tried and failed to respond to or tolerate available treatments.

Beyond the quantifiable change of PTSD symptoms, the degree to which MDMA supports the unfolding of a healing experience through neurochemical changes should be considered. Biochemically inducing a mental state more receptive to engaging in deep therapeutic processing could help to speed up symptom improvement or improve treatment outcomes for those resistant to other therapies. There is some evidence from nonclinical experiments that MDMA may increase neuroplasticity through BDNF-dependent mechanism (67), and otherwise alter brain activity in key networks for emotional-memory processing (30). Psychologically, MDMA may ease the challenge of recalling traumatic memories and feeling deeply into the associated emotions. Posttraumatic growth measured by the Posttraumatic Growth Inventory (PTGI), and personality shifts measured by the NEO Personality Profile have been observed after MDMA-assisted psychotherapy (43, 68). In addition, the importance of patient choice regarding therapy for PTSD has been pointed out, and MDMA-assisted psychotherapy may offer advantages in this area if it makes processing trauma less arduous (69).

Another recent meta-analysis paper, found no significant differences in benefits of pharmacological, psychotherapeutic, or the combination at the end of treatment, except at the last available endpoint during long-term follow-up, at which point psychotherapeutic treatments were significantly better than medications. In this analysis, the combined treatments, which included one MDMA-assisted psychotherapy trial, were slightly but not significantly more beneficial than psychotherapeutic treatments alone (66). Data from the other five phase 2 MDMA trials were not included, and the outcome from the MDMA trial was analyzed along with other medication-therapy combinations (e.g., SSRIs and CBT). Until MDMA-assisted psychotherapy is compared to trauma-focused therapies in a randomized trial, it is uncertain whether either approach is superior in terms of efficacy or tolerance. Though it may potentially have greater risks and increased likelihood of mild to moderate adverse events compared with non-drug therapies, MDMA has thus far demonstrated a favorable safety profile with limited administrations in clinical settings. Patient experience of each therapy, time to respond, and durability of response should be evaluated. Future research could also explore whether MDMA combined with existing manualized trauma-focused therapies potentiates PTSD symptom reduction.

## Status of MDMA Drug Development With Breakthrough Designation

BTD is intended to expedite the development and approval of promising treatments by allowing for more frequent interactions with the FDA, rolling review of documents, and the possibility for priority review (6 months rather than the normal 10-month review period) (1). BTD also receives an organizational commitment from the FDA with more guidance and involvement of FDA senior managers for efficient drug development.

After receiving BTD for this program, MAPS and the FDA also reached agreement under the Special Protocol Assessment (SPA) process for the design of two multi-site Phase 3 trials (MAPP1 and MAPP2) of MDMA-assisted psychotherapy for patients with at least severe PTSD. These two pivotal Phase 3 trials will enroll approximately 200-300 participants at sites in the USA, Canada, and Israel.

The pivotal Phase 3 trial started in November 2018. If Phase 3 trials produce significant confirmatory results and satisfactory safety profile, an application for marketing approval of MDMA-assisted psychotherapy for PTSD will be filed with the FDA. Filing of a New Drug Application is projected for 2021, with anticipated approval in 2022.

## Conclusion

It is anticipated that MDMA, with its unique pharmacological mechanisms combined with psychotherapy, has advantages over existing medications used as first-line PTSD treatments in terms of safety and side effect profiles, efficacy, and length of remission. PTSD is a chronic condition that afflicts a substantial number of individuals who do not adequately respond to available therapies and are at increased risk of suicide, other mental health conditions, cardiovascular disease, and cognitive impairment. Findings from both nonclinical and clinical studies support a novel mechanism by which MDMA amplifies the therapeutic effects of psychotherapy by a dynamic interaction of brain regions, and affiliated neurochemicals therein, known to be involved in fear extinction learning, memory reconsolidation, emotional processing, and cognition (30, 32, 39, 48, 70). With many apparent advantages over existing medications, including efficacy, tolerability, and duration of therapeutic effects, MDMA-assisted psychotherapy has the potential to favorably impact the lives of thousands who suffer from PTSD world-wide.

## Author Contributions

Concept and review design: LJ, AF, AE, BY-K, RD, and MM. Acquisition, analysis, or interpretation of data: LJ, AF, AE, BY-K, RD, and MM. Drafting of the manuscript: LJ, AF, AE, BY-K, RD, and MM. Critical revision of the manuscript for important intellectual content: LJ, AF, AE, BY-K, RD, and MM. Obtained funding: RD.

## Conflict of Interest Statement

AF received salary support for full time employment with MAPS PBC. LJ received salary support for full time employment with MAPS PBC. BY-K received salary support for full time employment with MAPS. AE received salary support for full time employment with MAPS PBC. MM received salary support from MAPS PBC as a clinical investigator and clinical trial medical monitor as well as for training and supervision of research psychotherapists. RD received salary support for full time employment with MAPS.

The Multidisciplinary Association for Psychedelic Studies (MAPS), a 501(c)(3) nonprofit organization, provided the MDMA and fully funded this study. MAPS Public Benefit Corporation (MAPS PBC), a wholly owned subsidiary of MAPS, was the trial organizer. MAPS and MAPS PBC assisted with study design; monitoring of study data; analysis, management, and interpretation of data; preparation, review, and approval of manuscript; and decision to submit the manuscript for publication. The funder had no role in the collection of data or conduct of the study.
